# Preparation of Carrier-Free Inhalable Dry Powder of Rivaroxaban Using Two-Step Milling for Lung-Targeted Delivery

**DOI:** 10.3390/pharmaceutics17050634

**Published:** 2025-05-09

**Authors:** Young-Jin Kim, Jaewoon Son, Chang-Soo Han, Chun-Woong Park

**Affiliations:** 1 P2KBio, Cheongju 28160, Republic of Korea; 7777ytrewq@p2kbio.co.kr; 2 MSAT/DP Team, GC Biopharma, Yongin-si 16924, Republic of Korea; sonjw96@gccorp.com; 3 Department of Pharmacy, Chungbuk National University, Cheongju 28644, Republic of Korea

**Keywords:** rivaroxaban, dry powder inhaler, pulmonary embolism, bead milling, jet milling, leucine

## Abstract

**Background/Objectives**: This study aimed to develop a dry powder inhalation (DPI) formulation of rivaroxaban (RVX) using a combination of bead milling (BM) and jet milling (JM) to enhance lung-targeted delivery for the effective treatment of pulmonary embolism while minimizing systemic exposure. **Methods**: A carrier-free DPI formulation of RVX was developed using sequential BM and JM, with L-leucine incorporated at various concentrations (1%, 5%, and 10%) as a force control agent. The formulations were characterized for particle morphology, size distribution, crystallinity, and thermal properties. The in-vitro aerodynamic performance was evaluated using a next-generation impactor, while ex-vivo studies assessed anticoagulant activity. Pharmacokinetic and tissue distribution studies were carried out in Sprague Dawley rats following intratracheal administration, and the effects of inhaled RVX were compared with those of oral administration. **Results**: The optimized BM-JM-5L formulation achieved a Dv50 of 2.58 ± 0.01 µm and a fine particle fraction of 72.10 ± 2.46%, indicating suitability for pulmonary delivery. The two-step milling effectively reduced particle size and enhanced dispersibility without altering RVX’s physicochemical properties. Ex-vivo anticoagulation tests confirmed maintained or improved activity. In-vivo studies showed that pulmonary administration (5 mg/kg) led to a 493-fold increase in lung drug concentration and 2.56-fold higher relative bioavailability vs. oral dosing, with minimal heart tissue accumulation, confirming targeted lung delivery. **Conclusions**: The two-step milled RVX DPI formulations, particularly BM-JM-5L with 5% leucine, demonstrated significant potential for pulmonary administration by achieving high local drug concentrations, rapid onset, and improved bioavailability at lower doses. These findings highlight the feasibility of RVX as a DPI formulation for pulmonary delivery in treating pulmonary embolism.

## 1. Introduction

Rivaroxaban, a non-vitamin K antagonist oral anticoagulant (NOAC), exerts its antithrombotic effect by selectively inhibiting coagulation factor Xa, thereby suppressing thrombin generation and, ultimately, preventing fibrin clot formation [1]. Rivaroxaban, a BCS Class II compound among NOACs, exhibits a high oral bioavailability, ranging from 80% to 100% [2,3]. Its oral formulation is approved for the treatment and prevention of deep vein thrombosis and pulmonary embolism (PE) in adults. However, the clinical profile of acute PE often necessitates emergency intervention, and oral administration of rivaroxaban, with an onset of action occurring 2.5 to 4 hours post-dose, may not be ideal in situations requiring rapid anticoagulation [4,5].

A dry powder inhaler (DPI) formulation of RVX may serve as a promising alternative for delivering the drug to the lungs, enabling both local and systemic therapeutic effects. Pulmonary administration enhances drug efficiency due to the lungs’ large surface area and thin epithelial barrier, which allows lower doses of RVX to achieve high local drug concentrations, a rapid onset of action, and relatively reduced systemic exposure [6,7,8]. Studies on DPI formulations of NOACs have been reported, including a micronized edoxaban-based DPI and an RVX formulation developed via spray-drying techniques for fast onset of action [9,10]. DPI formulations utilizing micronized drugs must maintain particle sizes in the low micrometer range to optimize inhalation efficiency. However, compared to submicronized (<1 μm) particles, micronized formulations generally exhibit slower dissolution rates, which may be further pronounced in the lungs due to their limited fluid volume [11,12]. Although spray-drying techniques can produce amorphous forms of RVX with enhanced dissolution rates, the physical instability of amorphous drug particles, especially in the absence of stabilizing polymers, raises concerns about recrystallization during storage and commercialization [13]. To overcome these limitations, a novel top-down two-step milling method was applied to prepare an inhalable RVX formulation.

A two-step milling method combining wet milling and jet milling was applied to develop an inhalable rivaroxaban (RVX) dry powder inhaler (DPI) formulation. This approach allows the use of starting materials and additives with particle sizes of several hundred microns without requiring separate premilling [14]. In the first step, wet milling was employed to produce submicronized RVX particles with increased surface area, aiming to enhance the dissolution rate of the drug [15,16]. Subsequently, the dried material underwent jet milling to achieve inhalable microparticles suitable for a carrier-free DPI formulation. This combined milling strategy addresses the limitations associated with using micronized particles alone, such as slow dissolution rates and potential instability of amorphous forms. By maintaining the crystalline state of RVX and achieving the desired particle size distribution, the two-step milling method facilitates the development of a stable and effective inhalable RVX DPI formulation.

Developing carrier-free dry powder inhaler (DPI) systems that minimize interparticulate interactions and circumvent blend uniformity issues, particularly in single-active pharmaceutical ingredient (API) formulations, has garnered increasing attention. Recent advancements in particle engineering have focused on reducing particle cohesion and enhancing dispersion efficiency, thereby facilitating the effective pulmonary delivery of high-dose drugs and improving overall therapeutic performance [17,18,19,20]. In such systems, additives such as L-leucine (LEU or L) and magnesium stearate are frequently employed as force control agents (FCAs) to improve powder dispersibility by reducing interparticulate cohesion and modifying surface characteristics [21,22,23]. Owing to its lubricating properties, LEU reduces surface energy, limits particle agglomeration, and improves drug dispersion [24,25]. This method not only eliminates the need for a separate individual JM step, it also maximizes surface coating with LEU and enhances particle size reduction.

This study aimed to develop a rivaroxaban (RVX) dry powder inhaler (DPI) formulation with rapid onset and high inhalation efficiency by employing a two-step milling process comprising wet milling and jet milling, incorporating L-leucine as a force control agent (FCA). The aerodynamic performance of the formulations was evaluated using a Next Generation Impactor (NGI), assessing the effects of the milling process and L-leucine addition on inhalation efficiency. Furthermore, the in-vivo distribution of RVX was investigated by directly measuring drug concentrations in the plasma, lungs, and heart of Sprague Dawley rats following pulmonary administration.

## 2. Materials and Methods

### 2.1. Materials

Rivaroxaban (RVX) was purchased from Alembic Pharmaceuticals (Gujarat, India). L-leucine and calcium chloride were purchased from Samchun Pure Chemicals Co., Ltd. (Pyeongtaek, Republic of Korea). Thrombin was purchased from Sigma-Aldrich (Darmstadt, Germany). Acetonitrile (HPLC grade) was purchased from Honeywell Burdick & Jackson, Ltd. (Muskegon, MI, USA). All other chemicals were of analytical grade and were used as received. All experiments were performed using Milli-Q distilled water (Merck, Kenilwroth, BJ, USA).

### 2.2. Preparation of Milled Rivaroxaban for Dry Powder Inhalation

The milled RVX dry powder formulations were prepared under the conditions listed in Table 1. The BM-only formulation was obtained by high-energy wet bead milling using PM-100 (RETSCH GmbH, Haan, Germany). In this process, 3 g of Raw-RVX was placed into a 50 mL jar along with 75 g of 1 mm zirconium beads and 20 mL of distilled water. The mixture was milled for five cycles. The resulting milled suspension was collected and dried in Oven (Model: ON-01E, JEIOTECH, Daejeon, Republic of Korea) at 50 °C for 12 hours to obtain a dry powder.

The JM-only formulation was prepared by air jet milling using an A-O Jet mill (JS Tech Co. Ltd., Sacheon, Republic of Korea). Three grams of Raw-RVX were subjected to milling under a grinding pressure of 0.45 MPa and a pushing pressure of 0.50 MPa.

The BM-JM formulation was prepared using a two-step milling system, with BM as the first step, followed by JM under the same conditions as the BM-only- and JM-only processes [14]. Furthermore, L-leucine-containing formulations (BM-JM-1L, BM-JM-5L, and BM-JM-10L) were prepared by adding 1%, 5%, or 10% leucine (*w*/*w*) relative to RVX during the BM process, based on previous reports suggesting that leucine concentrations within this range effectively improve powder dispersibility and aerosol performance in DPI formulations [14,26]. The mixtures were prepared using the same BM-JM process.

### 2.3. Physicochemical Characterization

#### 2.3.1. Morphology of Milled RVX Dry Powder

The morphologies and size distributions of the milled RVX formulations were characterized. Morphology was examined using a scanning electron microscope (SEM, Ultra Plus; Carl Zeiss, Jena, Germany). Samples were mounted on an aluminum plate using carbon tape, placed inside a Hummer VI sputtering device (Minneapolis, MN, USA), and then coated with platinum to discharge the particles, reaching a coating thickness of 600 Å.

The particle size distributions of the Raw-RVX and milled RVX formulations were determined using a Mastersizer 3000e (Malvern Panalytical, Great Malvern, UK), using the wet dispersion method, as dispersed in distilled water. The measurement principle is based on the simultaneous multi-angle detection of scattered light. Each measurement was conducted in triplicate, and the mean and standard deviation were calculated.

#### 2.3.2. X-Ray Diffraction (XRD)

The X-ray diffraction patterns of Raw-RVX, Raw-LEU, and BM-JM-5L were analyzed using a D8 Discover (Bruker, Billerica, MA, USA) at a wavelength of 1.54 Å. The 2θ scans were conducted between 5° and 60°.

#### 2.3.3. Differential Scanning Calorimetry (DSC)

The thermal responses of the pure substances (Raw-RVX and Raw-LEU), physical mixtures, and BM-JM-5L were analyzed using a DSC thermal analyzer system (DSC Q2000; TA Instruments, New Castle, DE, USA). The samples were accurately weighed, loaded into aluminum pans, and analyzed at temperatures ranging from 0 to 300 °C at a heating rate of 10 °C/min. The thermal response of the prepared sample was calculated using TA Universal Analysis Advantage software (version 5.2.6., TA Instruments, New Castle, DE, USA).

### 2.4. Particle Image Velocimetry

Particle dispersion characteristics of the milled RVX formulations were measured using a particle image velocimetry (PIV) system. Each sample containing 20 mg was loaded into an hydroxypropyl methylcellulose hard capsule (size 3). The RS01 device was mounted on a clear acrylic cube (200 × 200 × 200 mm). A flow rate of 60 L/min was maintained using a vacuum pump (Edwards, Lomma, Sweden). An 8 mV laser sheet beam (532 nm) was produced parallel to the device using a diode laser (LaserLab, Yongin, Republic of Korea). Particle behavior images were obtained at 2000 frames/s and 640 × 480 pixels using a high-speed camera (HAS-D71M, DITECT Corporation, Tokyo, Japan) positioned perpendicularly to the laser sheet plane [27]. The chamber was divided into 50×50 grids, and the images were processed using PIV analysis software (Flownizer 2D, DITECT Corporation, Tokyo, Japan). The particle velocities were obtained from the images between two frames using a combined recursive cross-correlation algorithm.

### 2.5. In-Vitro Aerodynamic Performance

Based on the USP Chapter 601 specification for aerosols, the aerosol performance of the milled RVX formulations was evaluated using a next-generation impactor (NGI, Copley Scientific Limited., Nottingham, UK) and RS01^®^ DPI device (Plastiape, Osnago, Italy). To prevent particle bounce and re-entrainment, the collection plates of the NGI stage were pre-coated with 3% silicone oil in hexane. Each sample containing 20 mg was loaded into an hydroxypropyl methylcellulose hard capsule (size: 3). A capsule was inserted into the RS01^®^, and the device was inserted into the mouthpiece of the induction port. Air was injected at a controlled flow rate of 60 L/min for 4 seconds. For NGI flow rate of 60 L/min, the aerodynamic cut-off diameters of each stage were determined as 8.06 μm, 4.46 μm, 2.82 μm, 1.66 μm, 0.94 μm, 0.55 μm, 0.34 μm, and 0.14 μm for stages 1–7 (S1–S7) and micro-orifice collector (MOC). The quantity of RVX remaining in the capsule and deposited onto each collection cup of the stage was measured using a validated High-performance liquid chromatography (HPLC). Analysis was performed using the HPLC system (Ultimate 3000 series HPLC system, Thermo Scientific, Waltham, MA, USA) and the Luna L11 column (250 mm × 4.6 mm, 5 μm) from Phenomenex Ltd. (Torrance, CA, USA). The mobile phase consisted of 55% acetonitrile in water (*v*/*v*) and was eluted at a flow rate of 1.2 mL/min [28]. The detection wavelength was set at 245 nm, and the injection volume was 20 μL. The calibration curve was linear in the range of 1.5 to 25 μg/mL (r^2^ = 0.9999). The emitted dose (ED), fine particle fraction (FPF), and fine particle dose (FPD) were calculated using the following equations:

Emitted dose [ED, %] = [initial mass in the capsule − final mass remaining in the capsule]/[initial mass in the capsule](1)

The fine particle fraction [FPF, %] = [Mass of particles at stage 2 to MOC]/[Mass of particles at all stages](2)

Fine particle dose [FPD, μg] = [FPF] × [Emitted dose, μg](3)

The mass median aerodynamic diameter (MMAD) and geometric standard deviation (GSD) were calculated from the drug mass deposition at the NGI stages using information from USP Chapter 601. All experiments were performed in triplicate.

### 2.6. Ex-Vivo Plasma Anticoagulation Effect Tests

The plasma anticoagulation assay was performed using a modified version of the method described by Rashid et al. [10]. Briefly, to ensure the robustness of the experimental setup, clot formation was validated under four different conditions: (1) no additives (only plasma), (2) 30 μL (0.2 M) of CaCl_2_ (+CaCl_2_), (3) 20 μL (0.5 U/mL) of thrombin (+thrombin), and (4) a combination of 30 μL CaCl_2_ and 20 μL thrombin (+CaCl_2_/thrombin). This validation allowed for a comprehensive assessment of the experimental reliability and ensured that the conditions were suitable for measuring anticoagulation effects. For the comparative analysis of RVX formulations, plasma (1 mL) was combined with 30 μL CaCl_2_ and 20 μL thrombin to induce clotting, following the validated method [29,30]. The Raw-RVX and formulation (BM-JM, BM-JM-5L) were dissolved in 1% dimethyl sulfoxide (DMSO) in phosphate-buffered saline (PBS) (pH 7.4) to a final concentration of 10 μg/mL as RVX. A blank control was prepared using 1% DMSO in PBS without RVX. In each experiment, 50 μL of the RVX solution or blank solution was added to 150 μL of the plasma mixture. The samples were incubated at 37 °C for 30 min for clot formation. Turbidity was measured at 405 nm using a SpectraMax ID3 plate reader (Molecular Devices, San Jose, CA, USA). The anticoagulation effect was evaluated by comparing the difference in absorbance between the initial time point and after 30 minutes for each sample. All experiments were performed in triplicate.

### 2.7. In-Vivo Study of Rivaroxaban

#### 2.7.1. Pharmacokinetic Studies of Rivaroxaban

The animals were treated and maintained in accordance with the principles of laboratory animal care approved by the Committee for Animal Experiments of Chungbuk National University (Cheongju, Republic of Korea). Sprague Dawley^®^ rats (males, 280–300 g) were purchased from SAMTAKO (Osan, Republic of Korea) and provided food ad libitum. During the experiment, the SD rats were kept under specified environmental conditions (20 °C, 40% relative humidity).

A pharmacokinetic (PK) study was conducted by administering RVX at a 5 mg/kg dose to the oral and inhalation groups. The oral RVX solution was initially prepared by dissolving RVX in DMSO, followed by dilution with an 80% PEG 400 solution to achieve a final concentration of 5% DMSO, resulting in an RVX concentration of 0.15 mg/mL [31,32]. The inhalation group (BM-JM-5L) was administered RVX as a dry powder via intratracheal instillation using an insufflator (DP-4, Penn-Century Inc., Philadelphia, PA, USA) via intratracheal instillation (ITI). Blood was collected from the orbital vein at 0.25, 1, 1.5, 2, 3, 4, 8, 12, and 24 h after administration. Plasma was immediately separated from the blood samples by centrifugation at 3000 rpm for 10 min. The drug concentration in plasma was determined using a validated method based on liquid–liquid extraction, which was modified from previously published protocols [33]. In brief, 100 μL of plasma was mixed with 50 μL of an internal standard (IS) solution (tadalafil dissolved in 100% acetonitrile, 500 ng/mL), followed by 1 mL of ethyl acetate. The mixture was vortexed at 2000 rpm for 1 min and centrifuged at 13,500× *g* for 10 min. The supernatant was transferred to a micro-centrifuge tube and dried at 50 ℃ under a nitrogen stream. The resulting residue was reconstituted in 200 μL of 45% acetonitrile and vortexed for 1 min. The final solution was centrifuged at 13,500× *g* for 10 min. The part of its supernatant was analyzed by HPLC-UV at 260 nm, using a Luna L11 column (250 mm × 4.6 mm, 5 μm) with a 1 mm × 50 mm guard column, and 5 μm-size particles (Waters Xterra MS C18, Waters Corp., Milford, MA, USA). The mobile phase consisted of 45% acetonitrile (ACN), and the flow rate was set to 1.0 mL/min. The column temperature was maintained at 40 μC, and an injection volume of 100 μL was used for the analysis. The method showed linearity between 12.5 and 500.0 ng/mL (r^2^ = 0.9991). All experiments were performed with n = 5.

#### 2.7.2. Lung and Heart Distribution of Rivaroxaban

Lung and heart tissue distribution was assessed by administering RVX at a 5 mg/kg dose to both the oral and inhalation groups. The rats were randomly assigned to five groups, treated with the drug, and sacrificed at predetermined time points (0.25, 2, and 4 h). To remove residual RVX drug particles that were not absorbed, the harvested lungs were washed three times using 5 mL of saline via the bronchoalveolar lavage fluid collection method. Subsequently, the lungs were placed in 10 mL of acetonitrile and homogenized at 20,000 rpm for 3 minutes using a homogenizer. The harvested heart tissues were placed in 10 mL of acetonitrile and homogenized at 20,000 rpm for 3 minutes using a homogenizer. The lung and heart homogenate were filtered through a 0.2 μm PVDF syringe filter (Whatman, GE Healthcare Co., Ltd., Chicago, IL, USA), and 450 μL of the filtrate was mixed with 550 μL of distilled water. The resulting mixture was analyzed using HPLC-UV under the same chromatographic conditions as those used for the plasma analysis (Section 2.7.1).

### 2.8. Statistical Analysis

Statistically significant differences were evaluated using one-way analysis of variance (ANOVA) followed by Tukey’s post-hoc test using SPSS version 23 (SPSS Inc., Chicago, IL, USA). Statistical significance was considered at *p* < 0.05 and *p* < 0.005.

## 3. Results and Discussion

### 3.1. Preparation and Characterization of Milled Rivaroxaban Dry Powder

**Figure 1:**
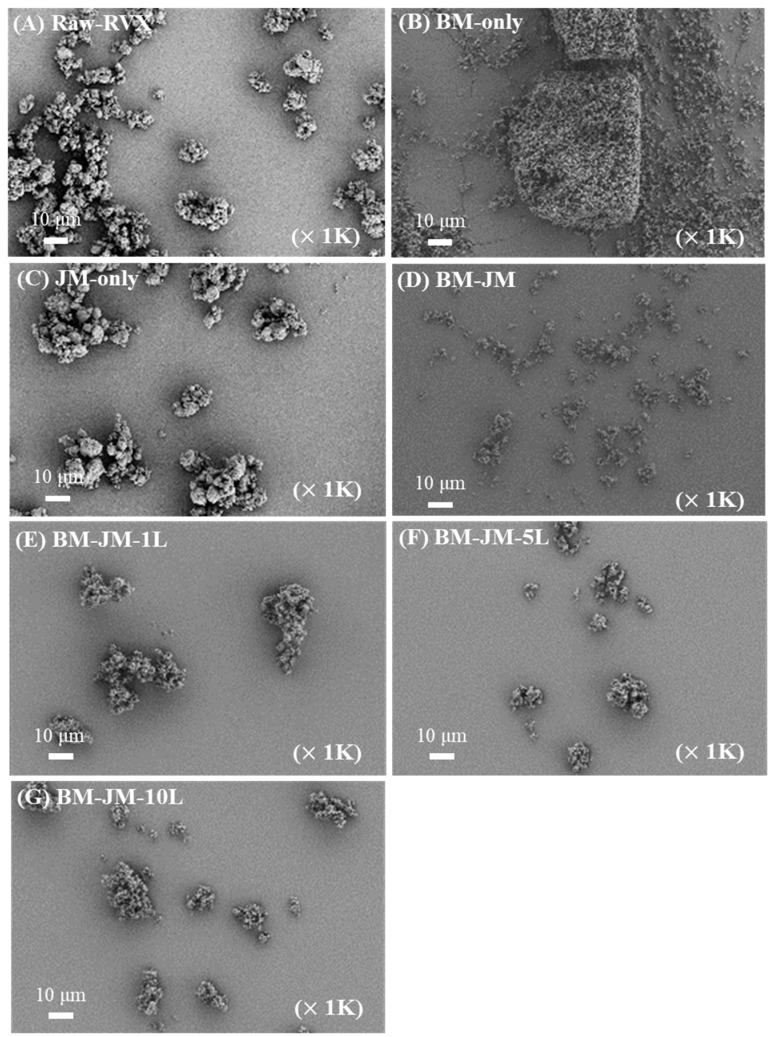
Scanning electron microscope images: (**A**) Raw-RVX, (**B**) BM-only, (**C**) JM-only, (**D**) BM-JM, (**E**) BM-JM-1L, (**F**) BM-JM-5L, and (**G**) BM-JM-10L.

SEM images of the Raw-RVX and milled RVX dry powder formulations are shown in Figure 1, and the particle size distribution values are shown in Table 2. Raw-RVX exhibited a particle size unsuitable for inhalation delivery, with Dv50 values measured at 8.27 ± 0.05 μm, indicating the need for further processing to achieve a suitable size range (<5 μm) [34,35]. SEM imaging revealed that the Raw-RVX particles displayed irregular shapes and sizes. BM only led to an unexpected increase in particle size, with Dv50 reaching 45.90 ± 0.36 μm. SEM images revealed that BM resulted in the formation of aggregated particles, likely owing to the hydrophobic nature of RVX, which increased its cohesiveness and promoted agglomeration [36]. Despite this aggregation, the primary particles were smaller than those of Raw-RVX. The jet milling (JM)-only formation slightly reduced the particle size, with a Dv50 value of 6.12 ± 0.04 μm, compared to the Raw-RVX. Despite this reduction, the particle size remained larger than required for inhalation. Additionally, the SEM images showed that the particle morphology and size of JM-only formulation were similar to those of Raw-RVX, with the primary particles exhibiting minimal milling. In contrast, the two-step milling (BM-JM) significantly reduced particle size, resulting in a Dv10 value of 0.89 ± 0.01 μm, Dv50 value of 2.84 ± 0.08 μm, and Dv90 value of 21.46 ± 1.75 μm. Dv10 and Dv50 values were below 5 µm, suggesting potential improvement in inhalation efficiency. In contrast, the Dv90 value indicated the presence of larger particles, likely resulting from aggregation, which may have a detrimental effect on aerosol generation in the inhalation formulations. The SEM images supported these findings, showing that the primary particles in the BM-JM formulation were smaller and more uniformly dispersed than those in the Raw-RVX and JM-only formulations. This suggests that while the BM process, as the first step, reduced the size of the primary particles, the subsequent JM process, as the second step, effectively dispersed these smaller particles, minimizing agglomeration and making the formulation more suitable for inhalation.

**Figure 2:**
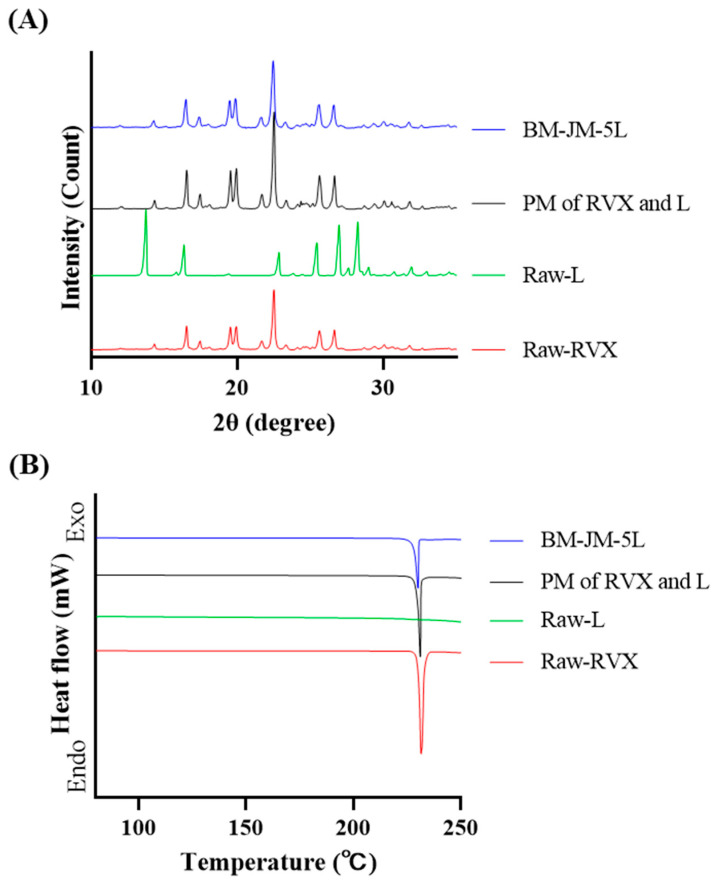
Physicochemical properties of milled RVX formulations with LEU: (**A**) X-ray diffraction patterns, (**B**) Differential scanning calorimetry thermograms.

To reduce the high cohesiveness of hydrophobic RVX, L-leucine was incorporated into the BM process as force control agent (FCA) at concentrations of 1%, 5%, or 10%. LEU is commonly used in DPI formulations to modify the surface of cohesive particles, improve particle dispersion, and reduce agglomeration. The effect of LEU on particle size distribution was evaluated, demonstrating that incorporation of 1% (BM-JM-1L), 5% (BM-JM-5L), and 10% LEU (BM-JM-10L) maintained Dv10 values below 1 µm and yielded Dv50 values ranging from 1.22 to 2.82 µm, which were comparable to those of the BM-JM formulation, as shown in Table 2. Notably, the addition of LEU resulted in a significant reduction in Dv90 values, with 7.39 ± 0.20 µm, 9.25 ± 0.08 µm, and 7.56 ± 0.08 µm for BM-JM-1L, BM-JM-5L, and BM-JM-10L, all of which were substantially lower than the Dv90 observed in BM-JM without LEU. It is thought LEU works by lowering the surface energy of dry powders, effectively coating the hydrophobic drug particles, which reduces particle–particle interactions, thereby decreasing their tendency to aggregate [14,37,38]. In addition, the SEM images further confirmed that the primary particles in the LEU-containing formulations were similar to those observed in BM-JM. The addition of LEU improved particle dispersion and reduced the formation of larger aggregates, enhancing the overall uniformity of the particle distribution. Following BM and subsequent JM with LEU, XRD and DSC analyses confirmed that no significant physicochemical changes occurred during the process, as shown in Figure 2. Unlike the physical mixture, the XRD pattern of BM-JM-5L did not show characteristic peaks of leucine. This is likely due to the reduction in diffraction intensity for both leucine and rivaroxaban caused by particle size changes during the bead milling process. Given the relatively low proportion of leucine in the formulation, its peaks may have been undetectable at the level observed in the physical mixture [39]. This indicates that the two-step milling process and the addition of LEU did not affect the crystallinity of RVX.

### 3.2. Comparison of Particle Dispersion and Aerodynamic Performance of RVX Formulations Prepared by Different Milling Methods

**Figure 3:**
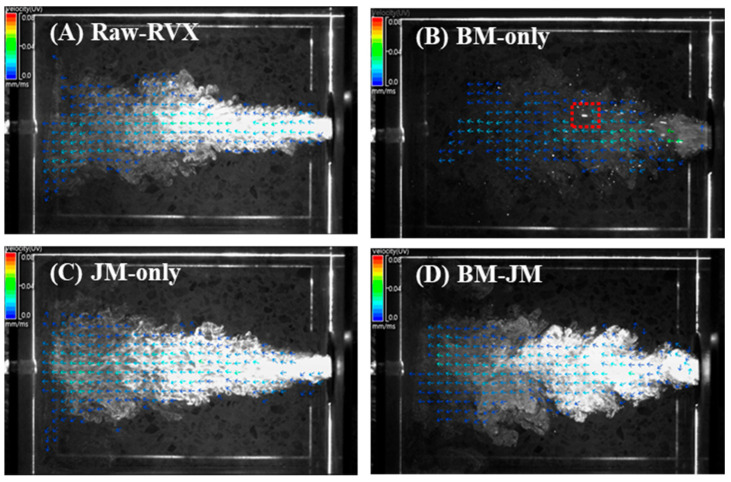
The particle dispersion characteristics of RVX were prepared using different milling methods. Velocity magnitude is visualized by a color gradient from blue (0 mm/ms) to red (0.08 mm/ms) and red dotted square indicates the aggregated particles.

The particle dispersion and aerodynamic performance of the milled RVX formulations prepared using different milling methods are shown in Figure 3 and Table 3. Particle dispersion was observed using the PIV system, and vector analysis results were obtained from the point of release to the arrival time within the side cross-plane of the particle dispersion. The Raw-RVX and JM-only formulations exhibited similar linear particle flow patterns, with the particles following direct paths and minimal dispersion. The arrival times for Raw-RVX and JM-only were 161.67 ± 46.12 ms and 158.67 ± 91.11 ms, respectively, with the mean of total particle speed recorded at 0.95 ± 0.08 mm/ms and 1.05 ± 0.02 mm/ms. Both formulations displayed comparable maximum speed at 3.00 ± 0.06 and 2.93 ± 0.08, respectively. In contrast, the BM-only formulation formed dispersed aerosols consisting of fine particles. However, some large particles were also observed, owing to aggregation. Consequently, although the mean speed was relatively low at 0.77 ± 0.08 mm/ms, the arrival time for the BM-only group was the shortest at 75.83 ± 10.10 ms, and the maximum speed was the highest, reaching up to 6.22 ± 0.09 mm/ms. This increases the likelihood of inertial impaction, which reduces the aerodynamic performance [40,41]. Meanwhile, the BM-JM formulation demonstrated a high degree of particle dispersion, with most particles moving in a vortex-like flow pattern similar to BM-only. However, BM-JM displayed a denser and more opaque appearance, indicating a higher particle concentration. The arrival time for BM-JM was 146.64 ± 25.90 ms, with the mean of the total particle speed of 1.00 ± 0.05 mm/ms and a maximum speed of 2.82 ± 0.11 mm/ms, showing no substantial differences from Raw-RVX and JM-only.

The aerodynamic performances of BM-only, JM-only, and BM-JM formulations were measured using next-generation impactor (NGI), as shown in Table 3. The BM-only exhibited an emitted dose (ED) of 69.69 ± 19.91% and a fine particle fraction (FPF) of 15.69 ± 1.91%. Neither the mass median aerodynamic diameter (MMAD) nor the geometric standard deviation (GSD) could be calculated (N/A) because more than 50% of the drug was deposited in stage 1. This suggested aggregated particles in the BM-only formulations resulted in poor aerosolization, leading to lower ED and FPF values. Although the milling process resulted in insufficient particle size reduction in the JM-only formulation, the absence of significant aggregation, unlike in the BM-only, contributed to an ED of 84.02 ± 6.17%, an FPF of 27.71 ± 2.49%, and a MMAD of 7.71 ± 0.03. Although particle size reduction was limited, the improved particle dispersion achieved through the JM process contributed to enhanced aerosol performance compared to the BM-only formulation. The BM-JM formulation exhibited an ED of 78.60 ± 1.48%, an FPF of 45.55 ± 49%, and a MMAD of 6.56 ± 1.31%. The significant improvement in FPF indicates that the reduction in the primary particle size and the effective dispersion of particles contributed to a more efficient aerosol performance. However, the ED was slightly lower than that of the JM-only treatment, likely because of the presence of aggregated particles formed during the BM process that were not fully dispersed to inhalable sizes during the subsequent JM process. This was further supported by the Dv90 value, which was larger in the BM-JM formulation than in the JM-only formulation, suggesting that some larger particles remained after the milling process, impacting the ED. Despite undergoing the BM-JM process, the MMAD remained above 5 µm, which is relatively large for an inhalable formulation [42].

### 3.3. Effect of Leucine Concentration on Aerodynamic Performance in Two-Step Milling Method

**Figure 4:**
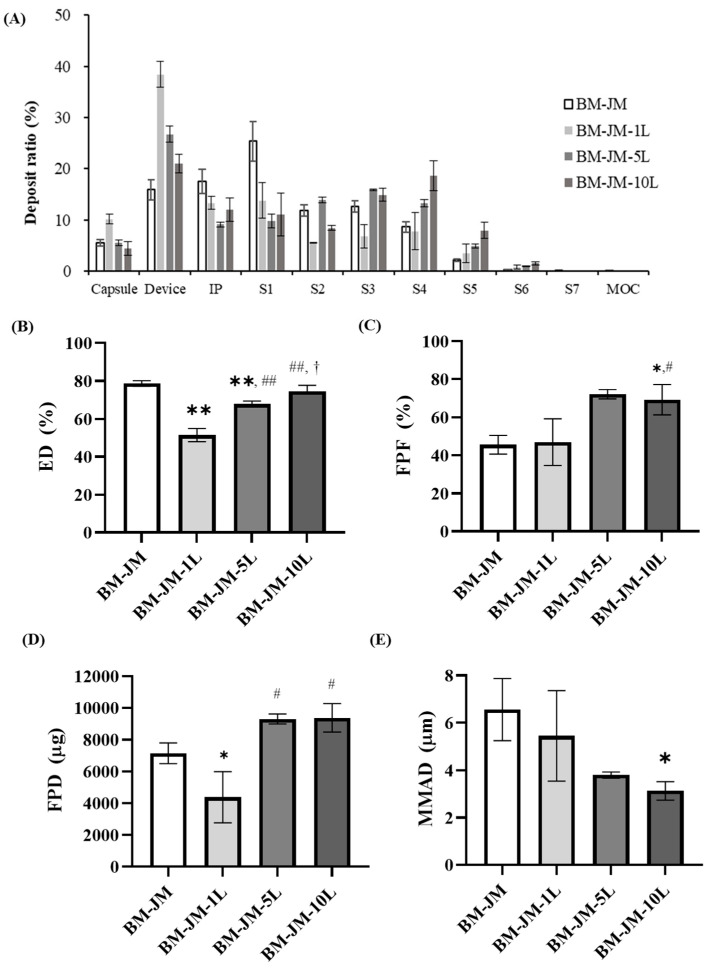
In-vitro aerodynamic performance characteristics of BM-JM, BM-JM-1L, BM-JM-5L, and BM-JM-10L (n = 3): (**A**) Deposit ratio of NGI stages, (**B**) Emitted dose (%), (**C**) Fine particle fraction (%), (**D**) Fine particle dose (μg), and (**E**) Mass median aerodynamic diameter. * ANOVA, *p* < 0.05 compared with BM-JM; ** ANOVA, *p* < 0.005 compared with BM-JM; # ANOVA, *p* < 0.05 compared with BM-JM-1L; ## ANOVA, *p* < 0.005 compared with BM-JM-1L; ^†^ ANOVA, *p* < 0.05 compared with BM-JM-5L.

In the formulations in which LEU was added during the BM process, the effects of LEU concentration on aerodynamic performance were evaluated, as shown in Figure 4. The ED values for BM-JM-1L, BM-JM-5L, and BM-JM-10L were 51.43 ± 3.46%, 67.77 ± 1.62%, and 74.57 ± 3.12%, respectively, which were lower than the ED observed in the BM-JM without LEU (78.60 ± 1.48%). The formulations of BM-JM and BM-JM with leucine tended to have lower ED values as Dv10 decreased. Similarly, in the milling method screening, formulations involving a BM process (BM-only and BM-JM) exhibited lower ED values than those involving JM only. In the leucine-containing formulations, the addition of leucine appeared to reduce the primary particle size during bead milling. This reduction may lead to stronger electrostatic interactions between the particles and the device, thereby decreasing the emitted dose [43,44].

In contrast, the FPF values for BM-JM-1L, BM-JM-5L, and BM-JM-10L are presented in Figure 4C and were 46.89 ± 12.25%, 72.10 ± 2.46%, and 69.22 ± 7.93%, respectively. The FPF for BM-JM-1L was comparable to that of the BM-JM without LEU (45.55 ± 4.90%), while BM-JM-5L and BM-JM-10L showed a significant improvement. An increase in FPF generally leads to improved delivery to the deep lungs, as reflected by the fine particle dose (FPD). For BM-JM-1L, the FPD was 4381.1 ± 1617.4 ng, which was lower than that of BM-JM without leucine (7151.7 ± 656.1 ng). In contrast, BM-JM-5L and BM-JM-10L showed significantly higher FPD values of 9306.2 ± 315.8 ng and 9366.7 ± 898.0 ng, respectively. These two formulations exhibited similar performance, with an approximately 1.3-fold increase in deep lung delivery compared with the leucine-free BM-JM formulation. The MMAD tended to decrease as the amount of leucine increased. This is considered to be a result of the addition of leucine, which likely influenced interparticle interactions and thereby improved powder dispersibility [19,23]. These results indicate that adding leucine during the BM process can reduce the emitted dose and enhance drug deposition in the deep lung by improving FPF. As no further improvement was observed beyond a 5% concentration, BM-JM-5L was selected as the optimal formulation in this study.

### 3.4. Ex-Vivo Plasma Anticoagulation Effect

**Figure 5:**
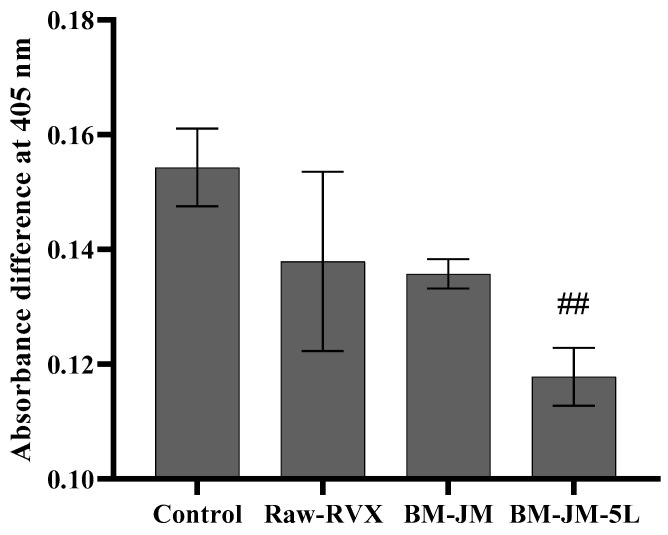
Absorbance differences at 405 nm relative to the non-treated condition after 30 min of incubation with RVX formulations treatment (n = 3). ## ANOVA, *p* < 0.005 compared to the control.

The plasma anticoagulation effect, measured as absorbance at 405 nm after 30 min of incubation, was assessed under various conditions to validate the experimental setup: Non-treated (1.149 ± 0.051), CaCl_2_-treated (1.239 ± 0.053), Thrombin-treated (1.211 ± 0.013), and combined CaCl_2_/Thrombin-treated (1.303 ± 0.007). These results confirmed that the combined CaCl_2_/thrombin-treated condition successfully induced clot formation, serving as a reliable control for subsequent anticoagulation studies. Comparative analysis of the Raw-RVX and RVX formulations (BM-JM and BM-JM-5L) was conducted by calculating the absorbance differences at 405 nm relative to the non-treated condition after 30 min of incubation and comparing these values with those of the control group (combined CaCl_2_/Thrombin-treated condition). The control group showed the highest absorbance difference (0.154 ± 0.007), indicating an absence of anticoagulant effect. As shown in Figure 5, the Raw-RVX produced a difference of 0.138 ± 0.016, confirming its expected anticoagulant activity. Both BM-JM (0.136 ± 0.003) and BM-JM-5L (0.118 ± 0.005) formulations demonstrated effective anticoagulation effects, suggesting that the BM and JM processes did not compromise the anticoagulation activity of RVX. Interestingly, BM-JM-5L showed a significantly lower absorbance difference than the blank control, suggesting an enhanced anticoagulation effect. It is possible that the presence of LEU in the BM-JM-5L formulation contributed to this observation, potentially through interactions between calcium ions and LEU. Previous studies have reported that co-administering heparin and leucine enhances anticoagulation via interactions between leucine and calcium ions. Similarly, co-administration with NOACs increases anticoagulation through the same calcium ion-mediated mechanism involving leucine [45,46]. Therefore, the introduction of LEU into the RVX inhalation formulation is expected to improve both the aerodynamic performance and the anticoagulation efficacy.

### 3.5. Pharmacokinetic Studies

**Figure 6:**
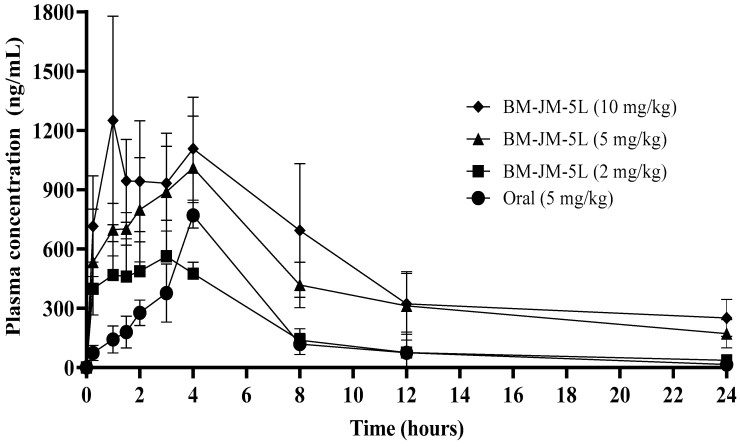
Pharmacokinetic profiles of plasma concentrations following oral and inhalation administration of RVX in Sprague Dawley (SD) rats (n = 5).

The pharmacokinetic profiles of oral RVX and inhaled BM-JM-5L formulations are shown in Figure 6 and Table 4. Oral administration was performed at a dose of 5 mg/kg, while the BM-JM-5L group was evaluated at 2, 5, and 10 mg/kg doses. At the earliest sampling time point of 0.25 h, the plasma concentrations observed following pulmonary administration of BM-JM-5L at doses of 2, 5, and 10 mg/kg were 398.75 ± 135.71, 534.22 ± 267.82, and 715.39 ± 255.02 ng/mL, respectively, which were approximately 5- to 10-fold higher than that observed with oral administration (73.74 ng/mL). This is expected to allow a rapid onset of action, similar to the previously reported amorphous rivaroxaban formulation studied by Alf Lamprecht, while maintaining the crystalline form of RVX [9]. Compared to the oral group, the relative bioavailability of the inhalation groups was significantly enhanced, showing 2.46-, 2.56-, and 1.61-fold enhancements for the 2, 5, and 10 mg/kg BM-JM-5L groups, respectively. These results suggest that pulmonary administration improves systemic absorption by bypassing first-pass metabolism and utilizing the lung’s large surface area, facilitating more rapid absorption and improved bioavailability compared to oral administration. Accordingly, a dry powder formulation may serve as a viable approach for emergency use or to achieve increased local drug availability in the treatment of pulmonary embolism [9]. The increased half-life (t_1/2_) in the inhalation groups, which correlated with higher doses, is likely due to the solubility limitations of RVX. As a poorly water-soluble compound, RVX may not dissolve rapidly upon deposition in the lungs, leading to saturation of the lung-lining fluid (10–20 mL/100 m^2^) at the local absorption sites. This saturation likely causes some dry powder to remain undissolved for an extended period, contributing to prolonged drug retention in the lungs. While this raises concerns regarding clearance by non-absorptive mechanisms (e.g., mucociliary escalators and alveolar macrophages) and potential pulmonary toxicity [47,48,49], direct pulmonary delivery of RVX offers a significant advantage by reducing the overall dose required to achieve therapeutic concentrations in the lungs. At these reduced inhaled doses, the risk of saturation in the lung lining fluid is minimized, decreasing the likelihood of undissolved particles remaining in the lumen [50,51]. To further address these concerns, future studies should aim to enhance the solubility of RVX, thereby promoting faster dissolution and absorption at the site of pulmonary deposition [52].

The superior pharmacokinetic performance of the inhalation groups is particularly evident in their ability to achieve comparable or higher lung-targeted drug concentrations at lower doses than oral administration. These findings suggest that pulmonary delivery may be a more effective strategy for treating pulmonary embolism (PE) as it allows for reduced systemic exposure while enhancing drug concentrations at the target site.

### 3.6. Lung and Heart Distribution

**Figure 7:**
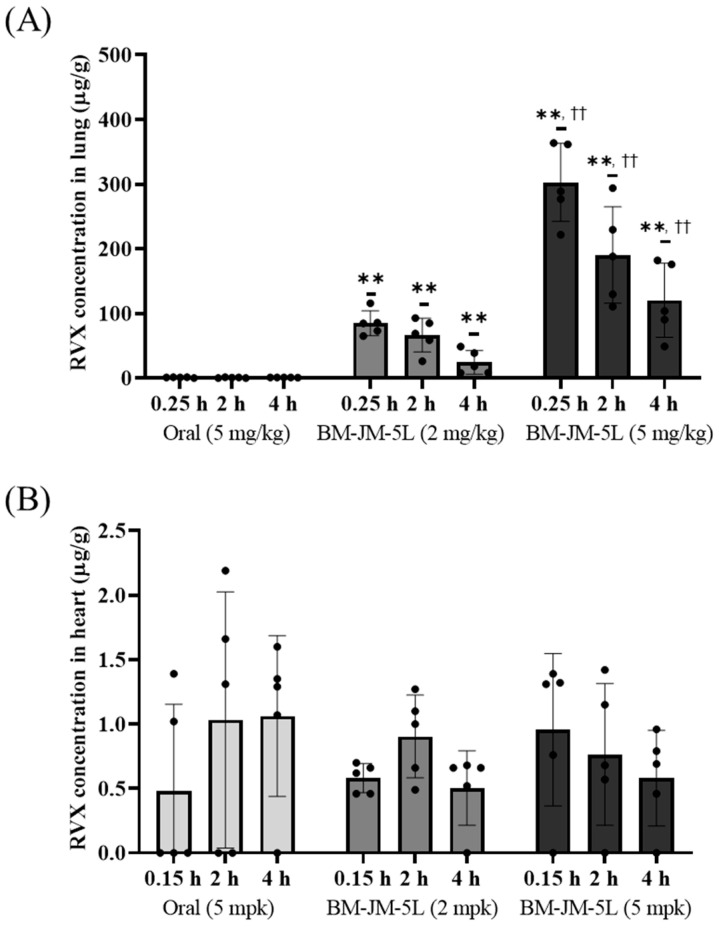
RVX concentration in lung and heart tissue at 0.25 h, 2 h, and 4 h post-administration in SD rats (n = 5). (**A**) Lung distribution, (**B**) Heart distribution. ** ANOVA, *p* < 0.005 compared with Oral (5 mg/kg); †† ANOVA, *p* < 0.005 compared with BM-JM-5L (2 mg/kg).

The lung and heart distribution profiles of oral RVX and inhaled BM-JM-5L formulations are shown in Figure 7. Both the 2 mg/kg and 5 mg/kg inhalation of BM-JM-5L groups exhibited significantly higher RVX concentrations in the lungs than the oral 5 mg/kg group. Following oral administration, lung concentrations of RVX were relatively low: 0.92 ± 0.52 μg/g at 0.25 h, 0.63 ± 0.62 μg/g at 2 h, and 0.97 ± 0.12 μg/g at 4 h. In contrast, the BM-JM-5L (2 mg/kg) showed significantly higher pulmonary concentrations despite the lower dose, with 84.90 ± 19.26 μg/g at 0.25 h, 66.37 ± 26.16 μg/g at 2 h, and 24.37 ± 18.43 μg/g at 4 h. The relative tissue uptake was 74-fold higher than that of the oral group. Similarly, BM-JM-5L (5 mg/kg) achieved even greater lung concentrations, 302.58 ± 60.37 μg/g at 0.25 h, 190.38 ± 74.61 μg/g at 2 h, and 120.24 ± 57.42 μg/g at 4 h, with a relative uptake exceeding 251-fold compared to the oral group. Notably, the concentrations observed at 0.25 h in both 2 mg/kg (*p* < 0.005) and 5 mg/kg (*p* < 0.005) inhaled groups were significantly higher than those in the oral group. In contrast, RVX concentrations in the heart tissue did not differ significantly between oral and inhaled formulations at either dose. At the observed time points, RVX concentrations in the heart tissue across all groups were generally below 1000 ng/mL, consistent with the systemic plasma levels. Additionally, when comparing the BM-JM-5L (5 mg/kg) inhalation group with the oral administration group at the same dose, the inhaled formulation delivered over 100-fold higher drug concentrations to the lungs relative to systemic exposure, highlighting its potential for targeted pulmonary delivery. These findings suggest that pulmonary administration of RVX allows for rapid and efficient drug delivery to the lungs, achieving a high local concentration at lower doses, which may contribute to a faster onset of therapeutic action. However, further evaluation in animal models is needed to determine whether the high pulmonary concentration of RVX can effectively prevent pulmonary embolism.

## 4. Conclusions

A carrier-free RVX dry powder inhaler formulation incorporating L-leucine as a fine particle control agent was successfully developed using a two-step milling process (ball milling followed by jet milling), achieving high aerosolization performance. The optimized BM-JM-5L formulation contained primary particles smaller than 1 μm and exhibited a high FPF of 72.10% in vitro. The crystalline structure and anticoagulation activity of RVX were retained, with leucine further enhancing its pharmacological effect. Pharmacokinetic studies demonstrated that pulmonary administration of the formulation enabled rapid systemic absorption compared to the oral route, along with a 2.56-fold improvement in relative bioavailability and a remarkable 493-fold increase in pulmonary drug deposition. These findings support the potential of the carrier-free RVX DPI formulation with leucine as a therapeutic option for acute pulmonary embolism. Despite these promising outcomes, the poor aqueous solubility of RVX remains a key limitation, which influenced the absorption rate. Future studies should explore solubility enhancement strategies and assess whether the observed pharmacokinetic benefits translate into improved therapeutic efficacy through comprehensive pharmacodynamic evaluation.

## Figures and Tables

**Table 1:** Process parameters of bead milling and jet milling.

Formulation			
Code	Component		Process
	Rivaroxaban	L-leucine	
BM only	3	-	Bead milling
JM-only		-	Jet milling
BM-JM		-	Bead and Jet milling
BM-JM-1L		0.03	
BM-JM-5L		0.15	
BM-JM-10L		0.3	
**Milling condition**			
Bead milling		Jet milling	
Bead	Zirconium beads 1 mm	Grinding pressure	0.45 MPa
Speed	650 rpm	Pushing pressure	0.50 MPa
Cycle	5 cycles Rotation time 12 min Interval time 3 min	Feed rate	40 Hz
Drying	50 °C, 12 hours		

**Table 2:** Particle size distribution of Raw-RVX and milled RVX formulations (Mean ± Stdev, n = 3).

Formulations	Dv 10 (μm)	Dv 50 (μm)	Dv 90 (μm)	Span
Raw-RVX	3.67 ± 0.00 ^##,††,!!^	8.27 ± 0.05 ^##,††,!!^	16.74 ± 0.40 ^##,††,!!^	1.58 ± 0.03 ^##^.^††,!!^
BM-only	4.82 ± 0.16 **^,††,!!^	45.9 ± 0.36 **^,††, !!^	86.88 ± 0.13 **^,††, !!^	1.78 ± 0.01 ^††,!!^
JM-only	2.16 ± 0.01 **^,##,!!^	6.12 ± 0.04 **^,##,!!^	13.94 ± 0.20 **^,##,††^	1.92 ± 0.02 **^,##,!!^
BM-JM	0.89 ± 0.01 **^,##,††^	2.84 ± 0.08 **^,##,††^	21.46 ± 1.75 **^,##,††^	7.21 ± 0.39 **^,##,††^
BM-JM-1L	0.58 ± 0.01 **^,##,††^	1.22 ± 0.01 **^,##,††^	7.39 ± 0.20 **^,##,††,!!^	5.57 ± 0.15 **^,##,††,!!^
BM-JM-5L	0.87 ± 0.01 **^,##,††^	2.58 ± 0.01 **^,##,††^	9.25 ± 0.08 **^,##,††,!!^	3.23 ± 0.03 **^,##,††,!!^
BM-JM-10L	0.89 ± 0.00 **^,##,††,!!^	2.82 ± 0.01 **^,##,††,!!^	7.56 ± 0.08 **^,##,††,!!^	2.35 ± 0.02 **^,##,!!^

** ANOVA, *p* < 0.005 compared with Raw-RVX; ^##^ ANOVA, *p* < 0.005 compared with BM-only; ^††^ ANOVA, *p* < 0.005 compared with JM-only; ^!!^ ANOVA, *p* < 0.005 compared with BM-JM.

**Table 3:** Particle dispersion and Aerodynamic performance of RVX prepared using different milling methods (Mean ± Stdev, n = 3).

Properties		Raw-RVX	BM-Only	JM-Only	BM-JM
PIV	Arrival time (ms)	161.67 ± 46.12	75.83 ± 10.10	158.67 ± 91.11	146.67 ± 25.9
	Mean speed (mm/ms)	0.95 ± 0.08 ^##^	0.77 ± 0.08 **^,††^	1.05 ± 0.02 ^##^	1.00 ± 0.05 ^##^
	Maximum speed (mm/ms)	3.00 ± 0.06 ^#^	6.22 ± 0.09 *^,††^	2.93 ± 0.08 ^##^	2.82 ± 0.11 ^##^
NGI	ED (%)	-	69.69 ± 19.91	84.02 ± 6.17	78.60 ± 1.48
	FPF (%)	-	15.69 ± 1.91 ^††^	27.71 ± 2.49 ^##^	45.55 ± 4.90 ^##,††^
	MMAD (μm)	-	N/A	7.71 ± 0.03	6.56 ± 1.31
	GSD	-	N/A	1.66 ± 0.02	2.61 ± 0.18

* ANOVA, *p* < 0.05 compared with Raw-RVX; ** ANOVA, *p* < 0.005 compared with Raw-RVX; ^#^ ANOVA, *p* < 0.05 compared with BM-only; ^##^ ANOVA, *p* < 0.005 compared with BM-only; ^††^ ANOVA, *p* < 0.005 compared with JM-only.

**Table 4:** Pharmacokinetic parameters of RVX for oral groups and inhalation groups in SD rats (Mean ± Stdev, n = 5).

Parameters	Oral (5 mg/kg)	BM-JM-5L (2 mg/kg)	BM-JM-5L (5 mg/kg)	BM-JM-5L (10 mg/kg)
t_1/2_ (h)	3.85 ± 1.29	6.27 ± 1.82	8.76 ± 1.54	10.46 ± 3.29
T_max_ (h)	4.00 ± 0.01	2.80 ± 0.45	3.80 ± 0. 45	3.80 ± 2.68
C_max_ (ng/mL)	770.26 ± 64.46	566.61 ± 134.22	1029.48 ± 256.54	1334.64 ± 465.97
AUC_0–24_ (ng·hr/mL)	4006.47 ± 1124.41	3945.45 ± 666.26	10,272.88 ± 2516.56 **	12,880.85 ± 565.1 **^,††^
CL/F (L/h/kg)	1.31 ± 0.28	0.45 ± 0.07	0.42 ± 0.11	0.30 ± 0.04
Relative BA	-	2.46	2.56	1.61

** ANOVA, *p* < 0.005 compared with Oral (5 mg/kg). ^††^ ANOVA, *p* < 0.005 compared with BM-JM-5L (2 mg/kg).

## Data Availability

The data presented in this study are available upon request from the corresponding author.

## Abbreviations

The following abbreviations are used in this manuscript:

PE	Pulmonary embolism
VTE	Venous thromboembolism
NOACs	Non-vitamin K antagonist oral anticoagulants
LEU or L	L-leucine
FCA	Force control agent
BM	Bead milling
JM	Jet milling
RVX	Rivaroxaban
DPI	Dry powder inhalation
SEM	Scanning electron microscope
XRD	X-ray diffraction
DSC	Differential scanning calorimetry
PIV	Particle image velocimetry
HPLC	High-performance liquid chromatography
NGI	Next-generation impactor
ED	Emitted dose
FPF	Fine particle fraction
FPD	Fine particle dose
MMAD	Mass median aerodynamic diameter
GSD	Geometric standard deviation
PBS	Phosphate-buffered saline
PK	Pharmacokinetic
ANOVA	Analysis of variance
AUC	Area under the curve
C_max_	Maximum plasma concentration
T_max_	Time to maximum concentration
T_1/2_	Half-life
CL/F	Clearance
BA	Bioavailability
